# High-power thin-disk lasers emitting beams with axially-symmetric polarizations

**DOI:** 10.1515/nanoph-2021-0606

**Published:** 2021-12-02

**Authors:** Marwan Abdou Ahmed, Frieder Beirow, André Loescher, Tom Dietrich, Danish Bashir, Denys Didychenko, Anton Savchenko, Christof Pruss, Marina Fetisova, Fangfang Li, Petri Karvinen, Markku Kuittinen, Thomas Graf

**Affiliations:** Institut für Strahwerkzeuge IFSW, Universität Stuttgart Fakultät 7 für Konstruktions- Produktions- und Fahrzeugtechnik, Pfaffenwaldring 43, Stuttgart 70569, Germany; Institute of Photonics, University of Eastern Finland, Joensuu, Pohjois-Karjala, Finland; Department of Physics and Mathematics, University of Eastern Finland, Joensuu, Finland; Institut für Technische Optik ITO, Universität Stuttgart Fakultät 7 für Konstruktions- Produktions- und Fahrzeugtechnik, Stuttgart, Baden-Württemberg, Germany

**Keywords:** azimuthal polarization, grating waveguide structures, nanograting-based polarization converters, radial polarization, thin-disk lasers

## Abstract

We present the intracavity generation of beams with radial polarization at an average output power of 750 W and an optical efficiency of 43% from a continuous wave thin-disk laser. Circular grating waveguide output couplers (GWOC) were used to select the radial polarization. The sensitivity of the polarizing function of the GWOC with regards to the fabrication tolerances is also analysed in details with a particular emphasis on the effect of the duty cycle and the geometrical profile of the gratings. Furthermore, we present the conversion of femtosecond laser pulses from linear to azimuthal polarization using a nanograting-based polarization converter. Azimuthally polarized beams with an average power of up to 850 W, a pulse duration of 400 fs and a pulse repetition rate of 1 MHz were generated in this way with a conversion efficiency of >90%.

## 1 Introduction

The properties of laser beams [1] such as the intensity distribution, wavelength, spectral bandwidth, as well as polarization are of particular importance for many applications in optics and laser technology. Most of the investigations and efforts in laser development were mainly focusing on increasing the brightness. Another aspect which has an important impact for many applications is the polarization of the laser beams. In interferometry for instance, a pure polarization state of a laser beam is required to avoid fading of the fringe contrast or measurement errors associated with polarization-dependent optical elements. The same applies for second-harmonic generation [2, 3], where the phase matching condition in the nonlinear crystal can only be achieved with a given and stable polarization state. In laser optics the most commonly used and known polarization states are linear and/or circular polarization. Linear polarization is usually determined by a Brewster plate, a conventional dielectric polarizer or the polarizing effect of resonator mirrors inside the laser cavity [4]. A quarter-wave plate can be used to convert the linear polarization to circular or elliptical polarizations.

**Figure 1:**
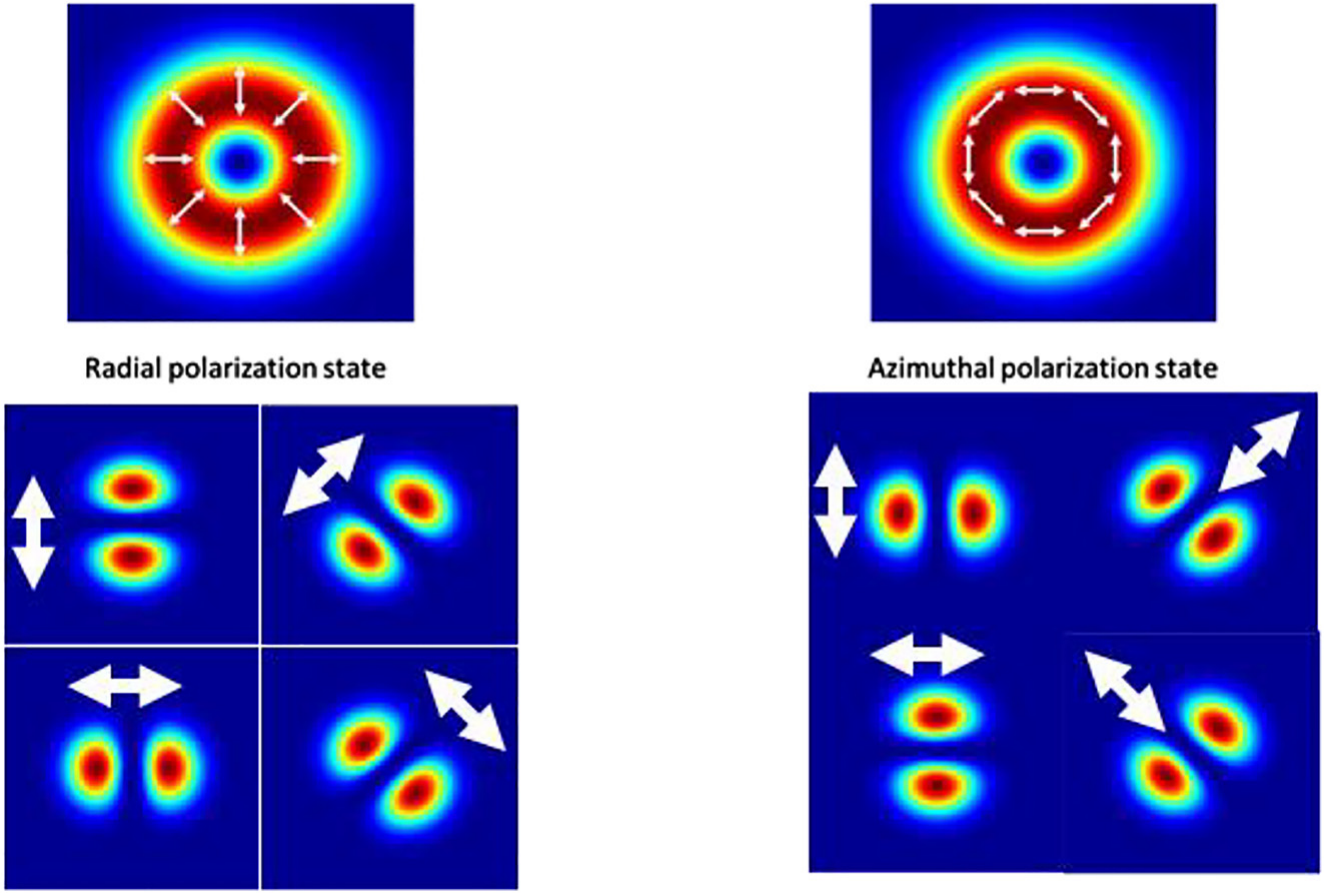
Calculated intensity distribution of beams with radial and azimuthal polarization states (top) together with the recorded intensity distribution transmitted through a polarization analyzer with its axis oriented at 0°, +/−45° and 90° (bottom).

In the past 45 years, the so-called axially-symmetric (inhomogeneous) polarization states and particularly beams with radial or azimuthal polarizations have attracted significant attention and an increasing interest in various fields of physics and laser-based manufacturing because of their specific and very attractive properties. These polarization-states are also known as radial, azimuthal (or tangential), and hybrid (mixture between radial and azimuthal) polarizations. Figure 1 illustrates the intensity distributions together with the orientation of the electrical field (represented by the white arrows) of the 2 aforementioned axially-symmetric polarization states. The electrical field of a radially polarized laser beam (TM_01_) always oscillates in radial direction (Figure 1(a)). In the azimuthal polarization state (TE_01_) the electrical field is oriented in tangential direction (Figure 1(b)).

Due to the polarization-dependent Fresnel-absorption an appropriate polarization distribution can significantly improve the efficiency of material processing. For metal cutting with CO_2_ laser it was predicted already theoretically by Niziev et al. [5] that radially polarized modes are preferable because of their significantly higher absorption in the kerf as compared to beams with linear or circular polarization [5]. For a linearly polarized Gaussian beam, it is shown that the energy is mainly absorbed in the center. For circularly polarized beams the absorbed energy corresponds to a Fresnel absorption averaged between *s* and *p* polarization states whereas for radially polarized beams a maximum of absorption was calculated over the entire cross-section. Using radial polarization therefore will lead to direction-independent cutting quality while exploiting maximum absorption over the entire cross-section. Thus, a higher cutting efficiency is expected when compared to commonly used circular polarization.

Venkatakrishnan et al. [6] and Meier et al. [7] reported on microdrilling experiments that clearly showed that azimuthally or radially polarized beams enhance the drilling efficiency depending on the drilling method and the processed material.

For solid-state lasers, radially or azimuthally polarized beams also have the advantage that they are not affected by thermally induced birefringence in homogeneously pumped cylindrical laser rods avoiding depolarization and bifocusing effects [8]; which usually limit the power range and the beam quality.

At low power levels, there are many other applications where radial or azimuthal polarization states showed a real benefit. Theoretical [9], [10], [11], [12], [13] and experimental [14], [15], [16], [17], [18], [19] investigations have been reported in the recent years on the strong longitudinal field enhancement obtained with a tight focusing of radially polarized light.

In ref. [14] it is shown that the smallest spot size ever experimentally demonstrated is obtained with radially polarized light. In this case the focus area is 0.16*λ* ^2^ whereas it equals 0.25*λ* ^2^ in the case of linearly polarized light. Such results open the way to several applications such as data storage [20], lithography [21], and resolution-enhanced microscopy [22], [23], [24], [25], [26], [27], [28], [29], [30], [31]. Particle acceleration [32], [33], [34], trapping or guiding [35], [36], [37], [38], orientation and probing of single molecules [39], [40], [41], and optical tweezers [42] also take advantage of the specific properties of beams with radial or azimuthal polarizations.

All these predictions as well as the first experimental demonstrations showing the benefits of radially and azimuthally polarized beams encouraged several scientific groups to investigate and to develop different approaches for the generation of such modes. Extra-cavity conversion [43], [44], [45], [46], [47], direct generation [48–53] and amplification [54–58] of beams with axially symmetric polarization have been extensively reported in the past few years.

In this paper, we present our latest achievements for the generation of beams with radial polarization using grating waveguide output couplers (GWOCs) where we could demonstrate up to 750 W of output power from a CW thin-disk oscillator. The conversion from linear to radial or azimuthal polarization of femtosecond pulses were furthermore realized using a nanograting-based polarization converter. Up to 850 W of converted average power were achieved with a conversion efficiency of >90% with pulse durations of <400 fs.

## 2 Intracavity generation of beams with radial or azimuthal polarization

### 2.1 Grating waveguide output coupler - GWOC: concept and design

**Figure 2:**
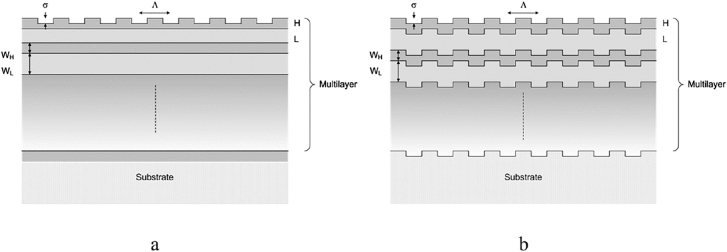
GWOC geometries with (a) grating defined in the topmost laser and (b) grating defined in all interfaces of the multilayer.

Grating waveguide output couplers or GWOC exploit the combination of a subwavelength grating and a partial reflector. Using a GWOC instead of a GWM (grating waveguide mirror) as reported in [51–53, 59–61] has the advantage that it can be combined with an SESAM in the resonator to generate ultrashort radially polarized pulses from a mode-locked oscillator. The two design geometries shown in Figure 2 are the most commonly used. Figure 2(a)) illustrates a GWOC where the grating is integrated in the topmost layer of the multilayer whereas Figure 2(b)) represents a multi-corrugated GWOC (i.e. where the grating is defined in all layer interfaces). This latter case is obtained by first etching the grating into the substrate and applying the coating afterwards.

**Figure 3:**
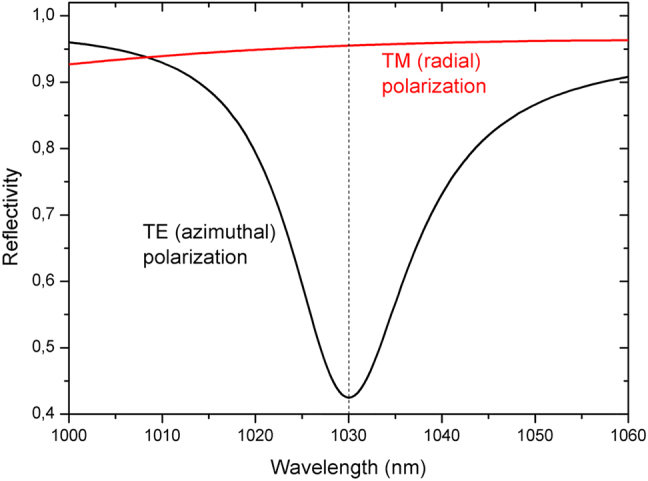
Calculated TE and TM reflectivity for a multiple corrugated GWOC.

As for the highly-reflective grating mirrors [51–53, 59–61], the polarizing mechanism is based on the coupling the radiation with the undesired state of polarization to leaky-modes of the waveguide formed by the coating stack. By this one can achieve a comparatively strong difference between the reflectivity for the radiation with the two polarization states. In a laser cavity, the modes with a polarization that leads to the lowest losses i.e. the highest reflectivity will oscillate. A GWOC designed, using modelling codes which are based on the modal method (also called RCWA – Rigorous Coupled Wave Analysis) [62], to obtain high reflectivity for TM polarized (corresponding to radial polarization when using a grating with circular lines) radiation and the lowest possible reflectivity for TE polarized (corresponding to azimuthal polarization when using a grating with circular lines) radiation is discussed in the following. A reflectivity difference of at least 20% is targeted in order to achieve sufficient discrimination of the two polarization states in a laser resonator. The multiple corrugation geometry was our first choice as it has shown a lower sensitivity to parameter deviations caused by manufacturing tolerances. The optimum reflectivity of the output coupler of typical Yb:YAG thin-disk lasers is in the range of 94–96%. The GWOCs were therefore designed to achieve similar values for the radially polarized radiation while ensuring a sufficient reduction of the reflectivity (>20%) for the azimuthally polarized radiation. A multilayer composed of 11 alternating Ta_2_O_5_/SiO_2_ quarter-wave layers (at 1030 nm) combined with a grating with a period of 890 nm and a groove-depth of 35 nm were found to meet the targeted performance. The calculated reflectivities for TE and TM polarized radiation are shown as a function of the wavelength in Figure 3. A reflectivity difference of >50% is obtained at a wavelength of 1030 nm while the calculated reflectivity for the TM (radial) polarized radiation is 95.5%.

**Figure 4:**
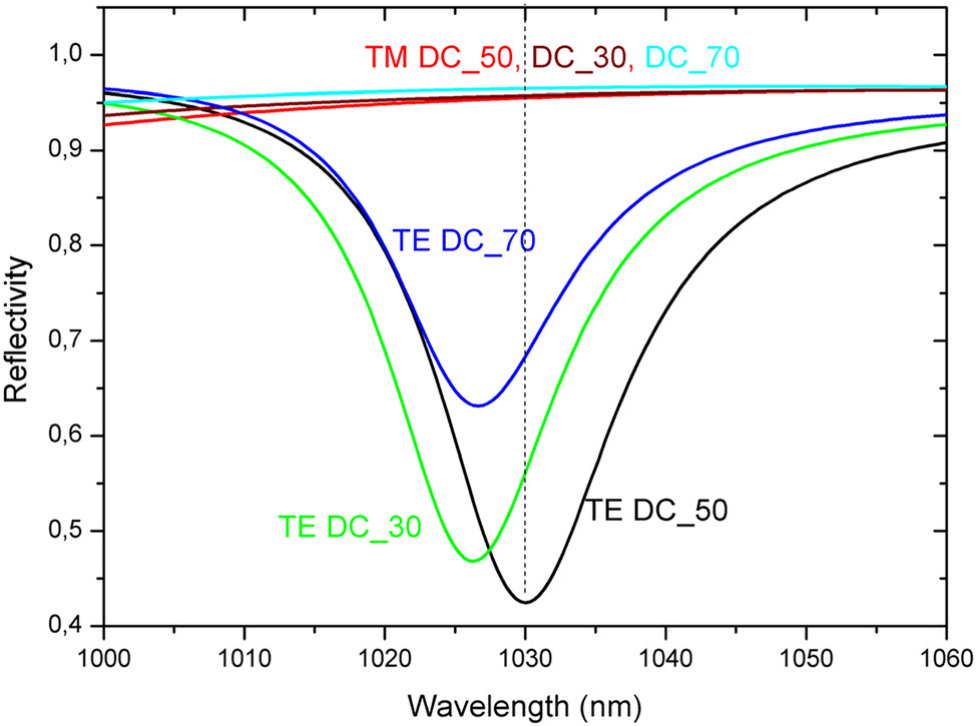
Calculated reflectivities for TE and TM polarized radiation of GWOCS with a grating duty cycles of 30, 50 (nominal design value) and 70%.

The tolerances to fabrication deviations were also analyzed. In the following we only focus on the effect of the grating’s duty cycle (DC) as this parameter if often reported by our fabrication partners to be the most challenging to control. Figure 4 shows the calculated reflectivities for TE and TM polarized radiation obtained with duty cycles of 30%, 50% (nominal design) and 70%. These values correspond to extreme cases as the fabrication can guaranty a DC value between 30% and 70%. As can be seen, the difference between the reflectivity for TE and TM polarized radiation at the central wavelength of our laser (i.e. 1030 nm) obtained with the three variants remains sufficiently high (>25%) to obtain an efficient intracavity generation of beams with radial polarization in a thin-disk laser. At the same time only a very small change of less than 1% of the reflectivity for the TM polarized radiation is observed. This latter remains within the values of 95–96% typically required for Yb:YAG thin-disk oscillators.

**Figure 5:**
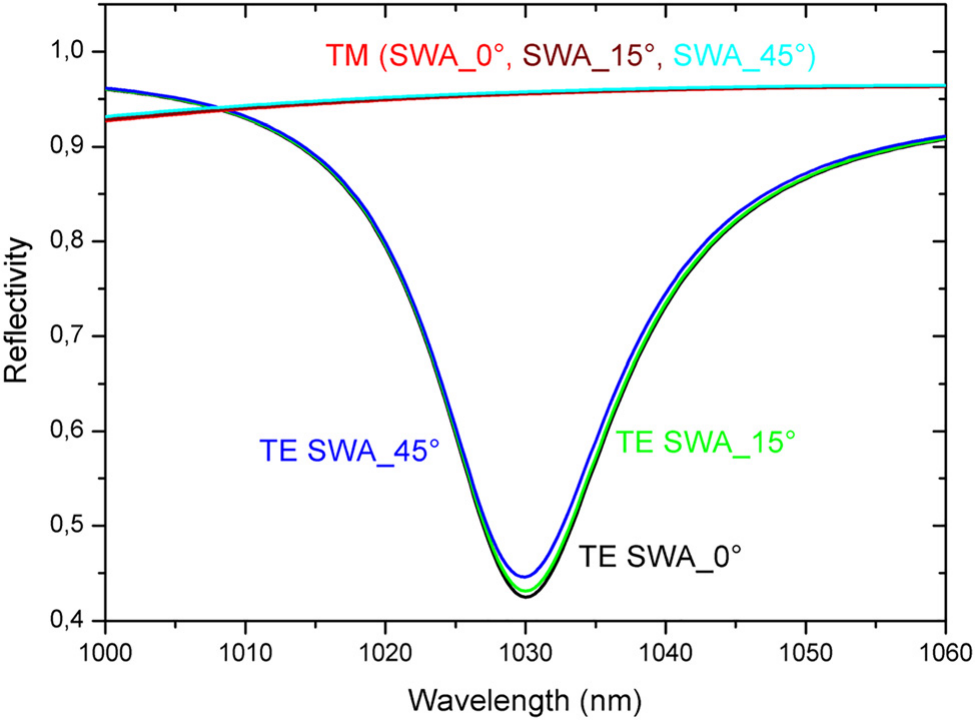
Calculated reflectivities for TE and TM polarized radiation for 3 different angles of the side walls (SWA) of the GWOC’s grating profiles.

Another parameter which is relatively difficult to control during the production is the grating’s profile. Nominal designs are often conducted assuming perfectly rectangular grating shapes. However, in reality the gratings exhibit trapezoidal profiles which, depending on the designs, may lead to more or less strong deviations of the optical performances of the grating device. In order to demonstrate the robustness of our design, a design analysis of the impact of the grating profile on the performances of our GWOCs is presented in the following. For the calculation we assumed 3 different profiles with side walls deviating by 0° (nominal rectangular profile), 15° and 45° from the vertical orientation. Once again these values correspond to extreme cases. Figure 5 shows the reflectivies for radiation with TE and TM polarization as calculated for the 3 cases. It is worth mentioning that the size of the top side of the trapezoid was kept to 50% of the period of the grating for all 3 designs. The profile was furthermore assumed to be identical over all interfaces (conformal profile). As can be seen, again very minor effects on the performance of the GWOC are observed. The low sensitivity to this parameter can be explained by the low aspect ratio (defined as the ratio of the grating depth by its linewidth and which is <10%) of the actual grating. This analysis is an additional confirmation of the robustness of the present GWOC design.

Another important aspect to consider here is the loss due to diffraction of the transmitted part of the radially (TM) polarized beam into the ±1st diffraction order. According to the design, this loss was found to be in the order of 1% (approx. 0.5% in each diffraction order). Consequently, based on the calculated reflectivity of 95.5% for radial (TM) polarization, around 22% of the power coupled out of the laser cavity is diffracted away from the useful laser beam. Nevertheless, it is worth mentioning that the GWOC designs we present here are based on a standard quarte-wave layer sequence and no specific optimization of the layer sequence was conducted in order to minimize the losses. In a future generation of the GWOCs the sequence of the layers will be optimized in order to reduce the diffraction losses for the transmitted beam which will enable the generation of radially or azimuthally polarized beams with a higher optical efficiency exceeding 50%.

### 2.2 Fabrication and characterization of the GWOC

**Figure 6:**
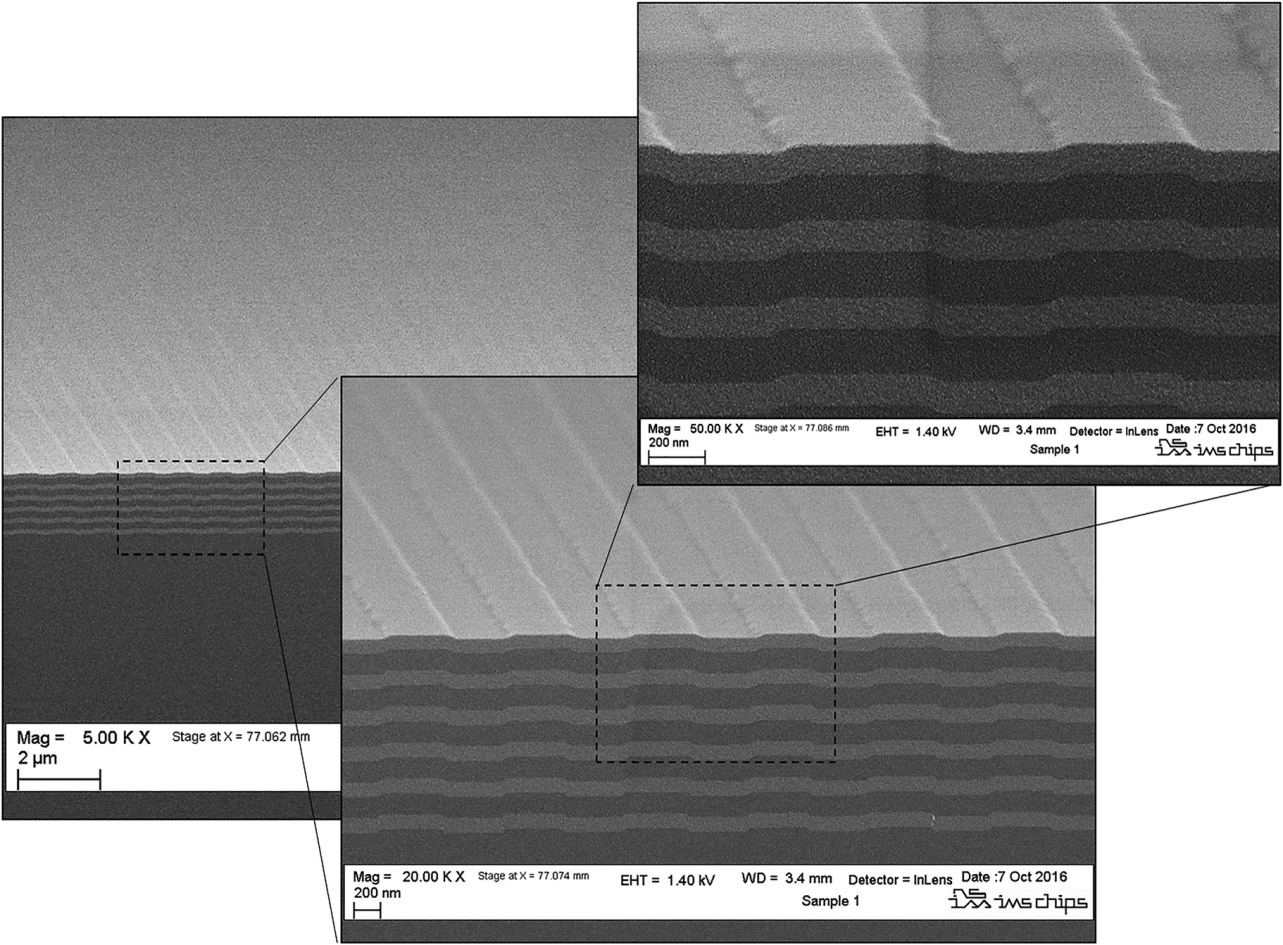
SEM image of a cross section through one of the fabricated GWOCs.

After the validation of its design, the GWOC was produced by means of Scanning Beam Interference Lithography [59, 63] followed by reactive ion etching of the fused silica glass substrate. Ion plating coating was applied in a sub-sequent step on top of the fabricated gratings to obtain the GWOC structure. A comparatively conformal grating across all layer interfaces is obtained thanks to the high-density ion plating coating, as revealed by the SEM image of a cross section of a similar GWOC (i.e. fabricated within the same run) shown in Figure 6. A groove depth of 31 nm was measured whereas the duty-cycle and the angles of the side walls are estimated to be approx. 55% and <45°, respectively.

**Figure 7:**
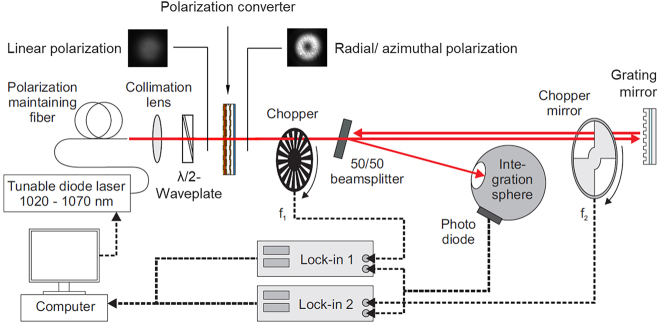
Setup for the spectroscopic measurement according to [58] modified by using a radially or azimuthally polarized beam to directly measure the reflectivities of the axially symmetric GWOCs.

Spectroscopic measurements of the fabricated GWOCs were conducted using the spectroscopic setup described in [59] with slight the modification shown in Figure 7 to directly measure the reflectivity of the circular grating of the GWOCs. A polarization converter [45] was introduced in the beam path to convert the polarization of the incident probe beam from linear to radial or azimuthal.

**Figure 8:**
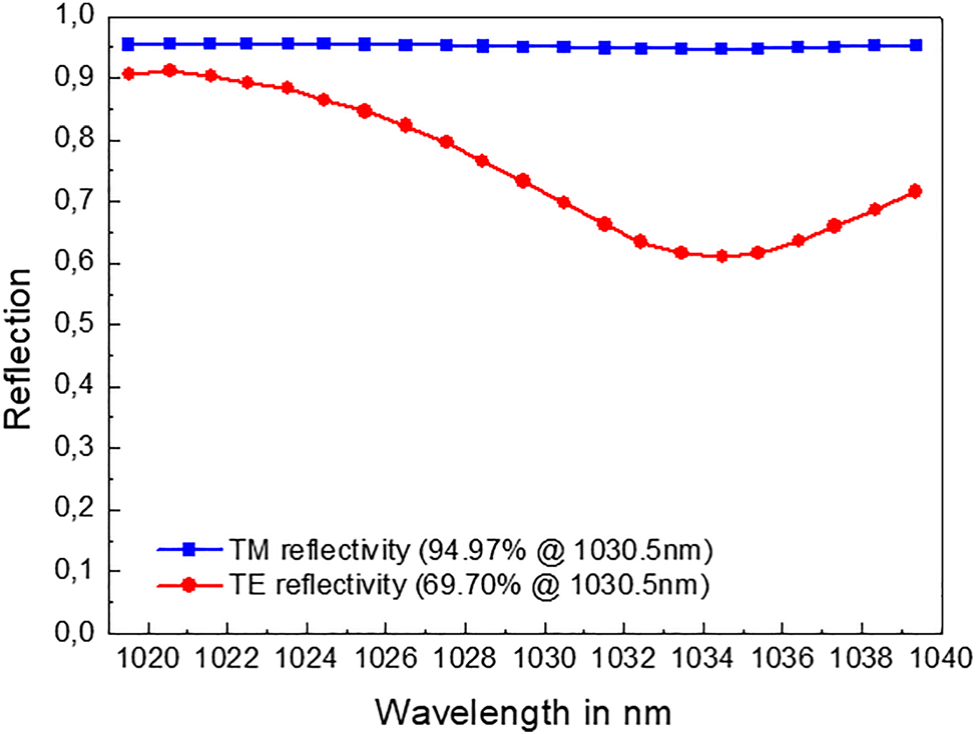
Measured radial (TM) and azimuthal (TE) reflectivities versus wavelength.

The TE and TM measured reflectivies of the GWOC are given in Figure 8.

**Figure 9:**
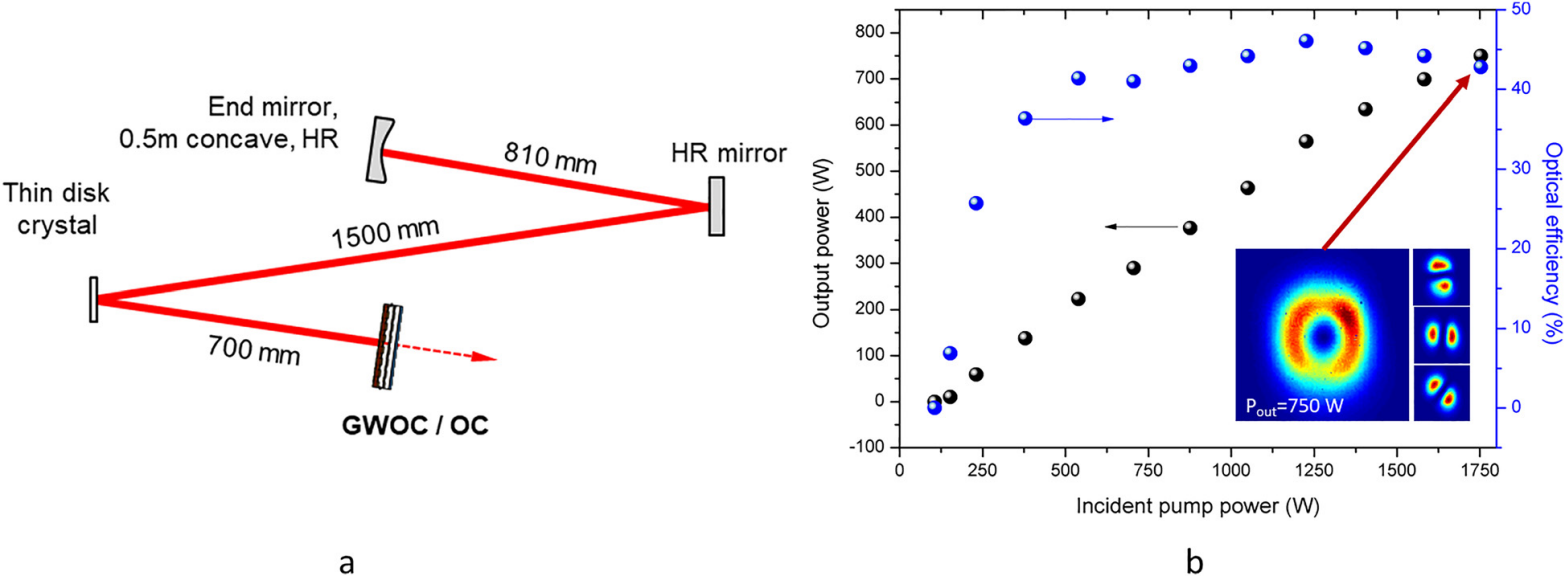
Experimental results obtained with the grating output coupler (GWOCS) with (a) Setup of the thin-disk laser oscillator using the GWOC for output coupling and (b) its laser performance.

Although the TE dip is red-shifted (assumed to be mainly due to a discrepancy between the refractive indices of the layers used in the design and those obtained by the fabrication), a sufficiently high reflectivity difference of >25% is achieved around the wavelength of 1030 nm to ensure proper laser operation using the GWOC as the output coupler of an Yb:YAG thin-disk laser oscillator. This latter was designed to support the oscillation of the LG_01_ mode (M^2^ = 2) and is schematically depicted in Figure 9(a). The Yb:YAG laser crystal was pumped at its zero-phonon absorption line with a wavelength of 969 nm in order to minimize the heat load inside the crystal (i.e. low quantum defect). Figure 9(b)) shows the measured output power and efficiency of the extracted radially polarized beam. Up to 750 W of average output power with an optical efficiency of approx. 43% was achieved with this concept. The beam quality factor was measured to be less than 2.5 at the maximum output power of 750 W.

The lower efficiency compared to the results obtained with a grating waveguide mirror used as the resonator’s end mirror rather than as its output coupler [61] can be explained by the additional loss ports resulting from the diffraction into the ±1st order experienced by the beam transmitted through the GWOC (cone in case of a circular grating). The far-filed intensity distribution of the emitted beam recorded at an output power of 750 W as well as the well separated lobes recorded behind a polarizing beam splitter (see inset of Figure 9(b)) indicatea high degree of radial polarization. The latter was measured using a camera-based polarimeter to be >(95 ± 1)%.

**Figure 10:**
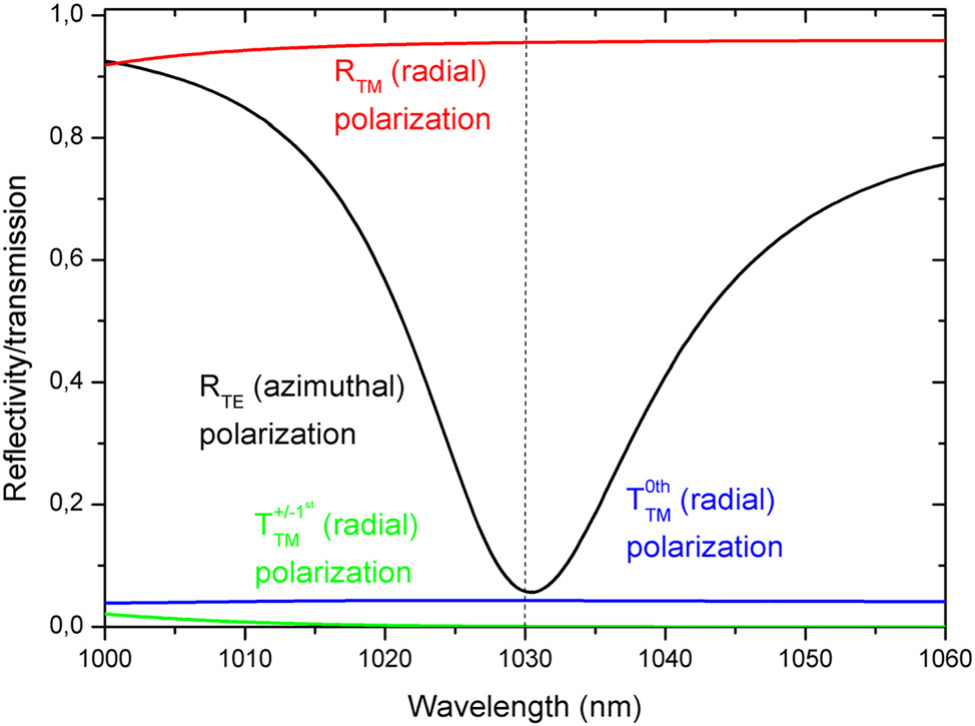
Calculated reflectivities (0th order) for TE and TM polarized radiation and transmissivities (0th and ±1st orders) for TM polarized radiation incident on the GWOC with the new design.

In order to further reduce the losses caused by the +/−1st diffraction orders a new GWOC design was developed. The structure is composed of 18 alternating Ta_2_O_5_/SiO_2_ layers coated on an etched substrate. The grating has a period of 880 nm and a groove-depth of 42 nm. The optimization consisted in the proper engineering of the thicknesses of the layers of the partial reflector. The calculated diffraction rate into the 0th reflected, 0th and +/−1st transmitted orders are shown in Figure 10. The calculated diffraction of TM polarized radiation into the +/−1st diffraction orders of about 0.1% were reduced by about a factor of 5 as compared to the ∼0.5% obtained with previous generation of GWOC described above. The difference of the reflectivities for TM and TE polarized radiation exceeds 85% with the actual design while the calculated reflectivity for the radiation with TM polarization amounts to approx. 95.6%.

A nanoimprint transfer followed by a reactive ion etching process was used to fabricate this new GWOC generation. A Master template with the above-mentioned grating period and duty-cycle was first defined in a silicon (Si) master template by e-beam lithography. The grating was then imprinted and etched in the fused silica substrate. Ion plating coating was applied in a subsequent step. After fabrication the GWOC was implemented in the setup for the reflectivity measurement depicted in Figure 7. The difference between the reflectivities for radial and azimuthal polarization was measured to exceed 65%. A high-polarization purity of the radially polarized beam is therefore expected.

**Figure 11:**
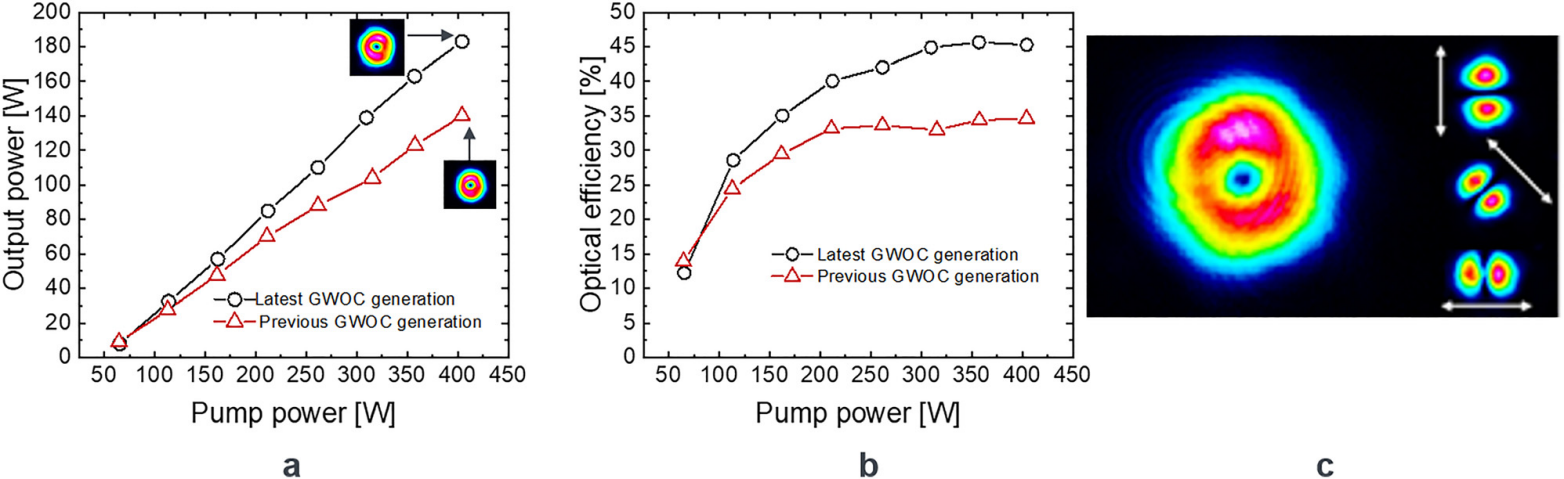
Laser performance comparing the two GWOC generations (left, middle) and qualitative analysis of the polarization of the output beam obtained with the new GWOC.

To demonstrate the efficiency improvement with the new generation of GWOCs the performance of the latter was first characterized in a continuous-wave thin-disk laser resonator with an output power in the order of 100 W. It is important to mention that the Yb:YAG thin-disk crystal was pumped at a wavelength of 940 nm, hence suffering from a higher quantum defect as compared to the zero-phonon pumping applied in the experiment described above. For better comparison the GWOC of the previous generation was also tested in this same resonator. The laser performance is shown in Figure 11 together with the qualitative analysis of the polarization of the beam obtained with the new GWOC. As can be seen an output power of approximately 185 W at an optical and slope efficiencies of around 45% and 53% were achieved using the latest generation of GWOCs. This corresponding to an increase in the optical and slope efficiencies by approx. 10 and 14 percentage points respectively in comparison to the previous GWOC generation. The degree of radial polarization was measured to be >(96 ± 1)%.

**Figure 12:**
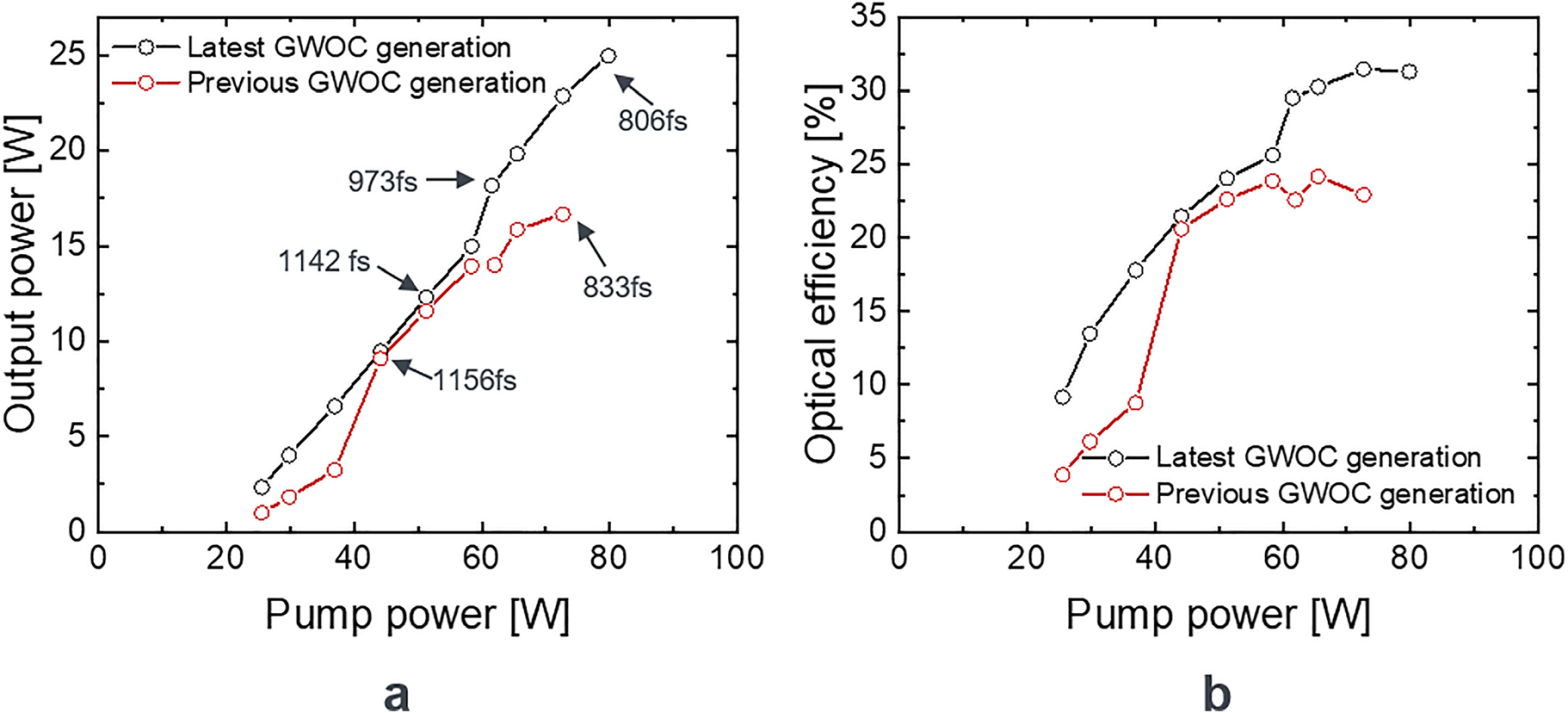
Laser output power (a) and optical efficiency (b) using the GWOCs in a mode-locked thin-disk laser.

A comparison of the previous and the latest generation of GWOCs used in a mode-locked laser at moderate average power is shown in Figure 12. With the latest generation of the GWOC, an average output power of 25 W with an optical efficiency of 31.3% was achieved. Using the previous generation of GWOCs under exactly the same conditions led to an average output power of only 16.7 W with an optical efficiency of 22.9%. Hence, the latest generation of GWOCs enabled an increase of the optical efficiency by more than 8 percentage points. At the maximum power of 25 W, the pulse duration was measured to be 806 fs.

Further experiments at significantly higher powers in both continuous-wave and mode-locked operation including a detailed design analysis of this new generation of GWOCs are currently in progress and will be reported on at a later date.

## 3 Extra-cavity conversion of linear to radial or azimuthal polarization

**Figure 13:**
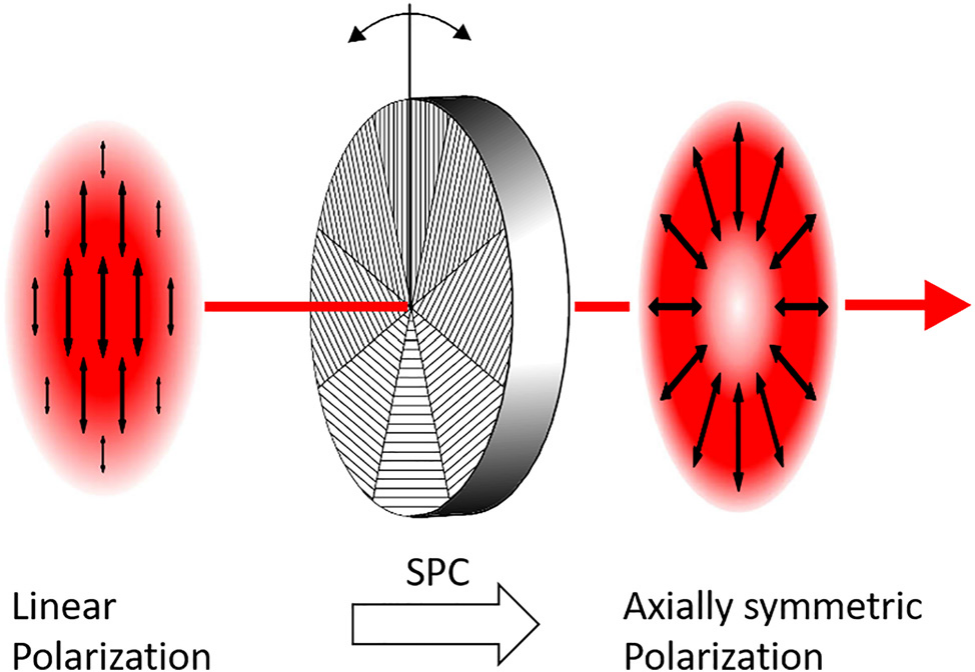
Principle of the conversion of a linearly polarized beam into on with axially symmetric polarization (example with 8 segments of half-wave plates). SPC: segmented polarization converter.

Ultrafast lasers often emit beams with linear polarization. The most obvious way to take benefit from beams with radial or azimuthal polarizations is therefore to use segmented polarization converters (SPC). The polarization conversion mechanism of the SPC is based on form birefringence, i.e. a local difference between the phase velocities of the polarization components normal and parallel to the orientation of the grating lines. The SPC locally rotates the polarization of the incoming beam such that it corresponds to the desired polarization state. To achieve a rotation of a linear polarization (half-wave plates effect), a *π* phase shift between the orthogonal polarization states is needed. The most commonly established converters consist of segmented half-wave plates (8, 12, or even more segments of half-wave plates) to convert a linearly polarized incident beam to radially or azimuthally polarized beams, as schematically shown in Figure 13.

Materials with natural birefringence such as quartz or MgF_2_ are often used to compose the half-wave segments. Alternatively, form-birefringence induced by the implementation of sub-wavelength gratings (nanogratings) in an isotropic material such as fused silica represents a valuable approach as it has already shown promising results [45]. The orientation of the grating lines corresponds to the fast axis of such wave plates.

**Figure 14:**
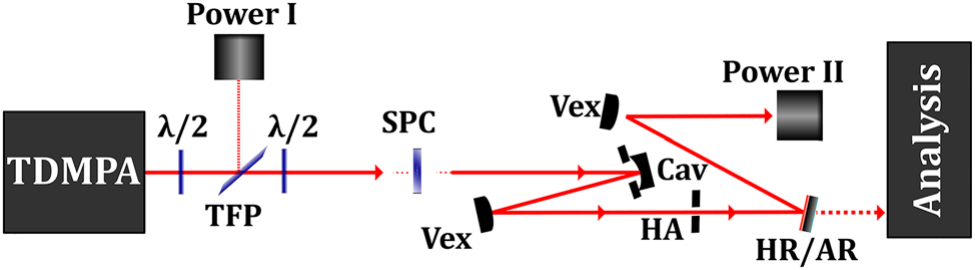
Experimental setup for the conversion of the linear polarization of a beam into radial or azimuthal polarization.

Nanogratings written into the volume of the substrates by femtosecond laser pulses at the Workshop of Photonics [64] were used to setup the segmented polarization converter (SPC), which was then deployed to convert a high-power (kW-class) beam of femtosecond pulses delivered by a thin-disk multipass amplifier (TDMPA) similar to the one presented in [65–67]. The setup for the conversion experiment is sketched in Figure 14. A combination of half-wave plates and a thin-film polarizer were used to adjust the polarization as well as the power incident on the segmented polarization converter. The beam diameter on the SPC was set to 3.5 mm. Beam cleaning apertures were introduced in order to filter out the scattered light caused by the central zone and interfaces between the segments of the SPC. The converted beam was analyzed behind a high-reflection (HR) mirror whose rear side was anti-reflection (AR) coated.

**Figure 15:**
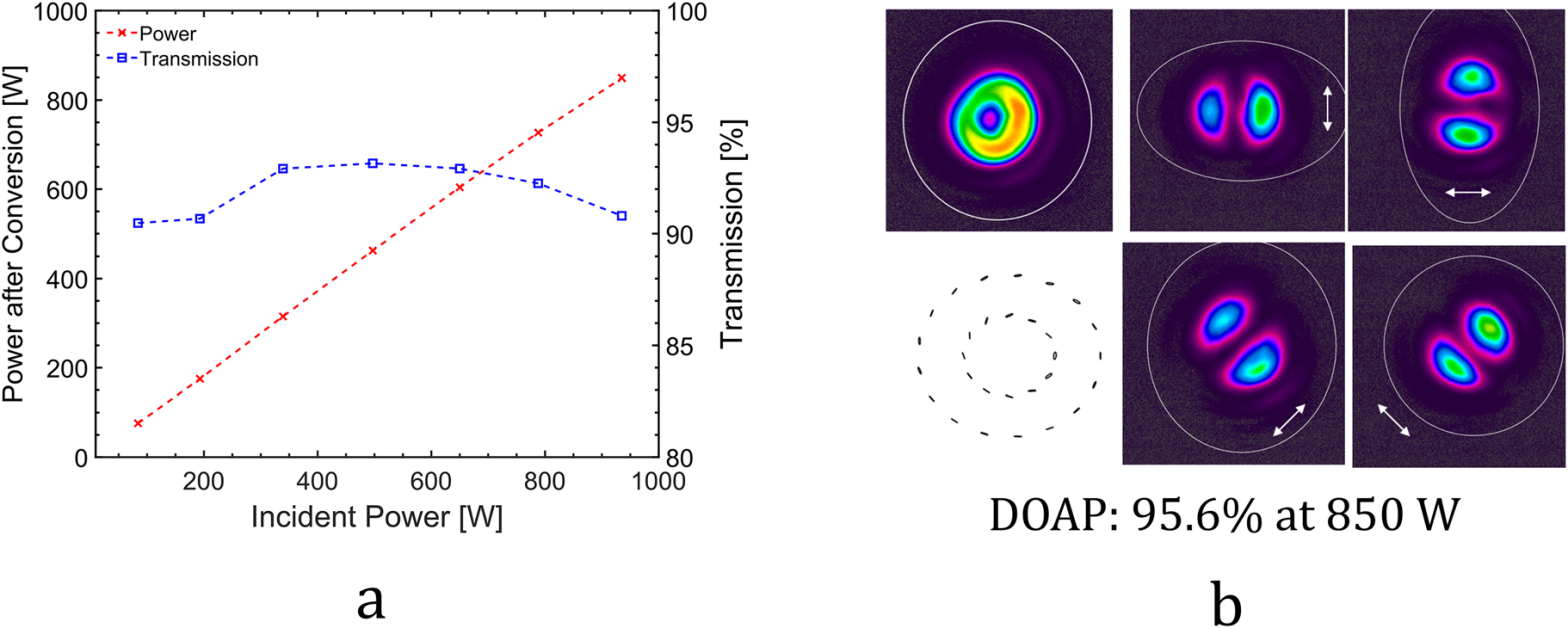
Experimental results obtained with the segmented polarizaton converter (SPC) with (a) Measured power transmission through the polarization converter and cleanup; (b) recorded intensity distribution of the converted azimuthally polarized beam without and with a polarization analyzer in the beam path. The polarization ellipses measured in the cross section of the beam are shown on the bottom left of (b).

The TDMPA delivered up to 950 W of average output power in a linearly polarized beam with femtosecond laser pulses. The pulse repetition rate was 1 MHz. The beam propagation factor *M* ^2^ and the duration of the incident pulses were measured to be < 1.3 and < 400 fs, respectively. It is worth mentioning that the conversion to both radial and azimuthal polarization was investigated. The change of the transmitted beam from radial to azimuthal polarization is simply achieved by 90° rotation of the incident polarization. Within the experimental accuracy, the same performance was obtained in both cases. Only the results obtained for the conversion to azimuthal polarization is therefore discussed in the following. The measured transmitted power and conversion efficiency are shown in Figure 15(a). A power transmission of at least 90% was measured (after the beam cleanup) in the whole power range. The observed variation by a few % is mainly attributed to the residual reflection due to the non-AR-coated SPC facets. A maximum power of 850 W was obtained in the converted and cleaned-up beam. The analysis of the polarization of the transmitted beam is shown in Figure 15(b). The degree of radial/azimuthal polarization was measured to exceed 95% using our 2D-polarimeter. The polarization ellipses depicted in Figure 15(b) (bottom-left) are shown to illustrate the polarization state at 2 different radii over the beam cross-section. The measurements are however recorded for each single pixel.

The beam propagation factor *M* ^2^ was measured to amount to less than 2.5 in the whole power range. The pulse duration in the converted beam was estimated to be similar to that of the incident beam i.e. <400 fs.

## 4 Conclusions

In conclusion we have shown two methods capable of generating high-power beams with axially symmetric polarizations. One consists in a robust grating waveguide output coupler (GWOC) used for intracavity generation of beams with radial polarization with which up to 750 W of continuous-wave output power was demonstrated. A detailed design analysis was conducted to study the impact of fabrication tolerances on the optical performance of the GWOC. These revealed a comparatively low sensitivity of the reflectivity of the GWOC to deviations of the shape and the duty-cycle of the grating. The performance of the GWOC was further confirmed by their spectroscopic and intracavity characterizations. A new GWOC design was finally proposed in order to reduce the losses for the transmitted radiation by the power leakage into the +/−1st diffraction orders. First laser experiments at moderate powers showed an increase of the optical efficiency by approx. 10% over the previous GWOC design. This opens the way for further power scaling including mode-locked operation. We also reported on the generation of radially and azimuthally polarized beams of pulses with a duration of < 400 fs at a repetition rate of 1 MHz with an average power of 850 W using a nanograting-based polarization converter. This latter exhibited a conversion efficiency of more than 90% over the whole power range available in the experiment. As no indication of a thermally induced roll-over was observed, it is expected that the device can handle even higher powers.

In summary, it was shown that depending on the application and needs both intracavity or extra-cavity solutions can provide valuable approaches to generate high-power continuous-wave or mode-locked beams with radial or azimuthal polarizations.
